# Performance of Organic Electrochemical Transistors with Ionic Liquid Crystal Elastomers as Solid Electrolytes

**DOI:** 10.1021/acsami.4c06608

**Published:** 2024-09-26

**Authors:** Arwa Alyami, Michael Skowrons, Kelum Perera, Björn Lüssem, Antal Jákli

**Affiliations:** † Department of Physics, Kent State University, Kent, Ohio 44242, United States; ‡ Advanced Materials and Liquid Crystal Institute, Kent State University, Kent, Ohio 44242, United States; § Institute for Microsensors, Microactuators, and Microsystems (IMSAS), University of Bremen, Bremen 28359, Germany; ∥ MAPEX Center for Materials and Processes, University of Bremen, Bremen 28359, Germany

**Keywords:** organic electrochemical transistor, liquid crystal, ionic liquid crystal elastomer, lateral geometry, wearable electronics

## Abstract

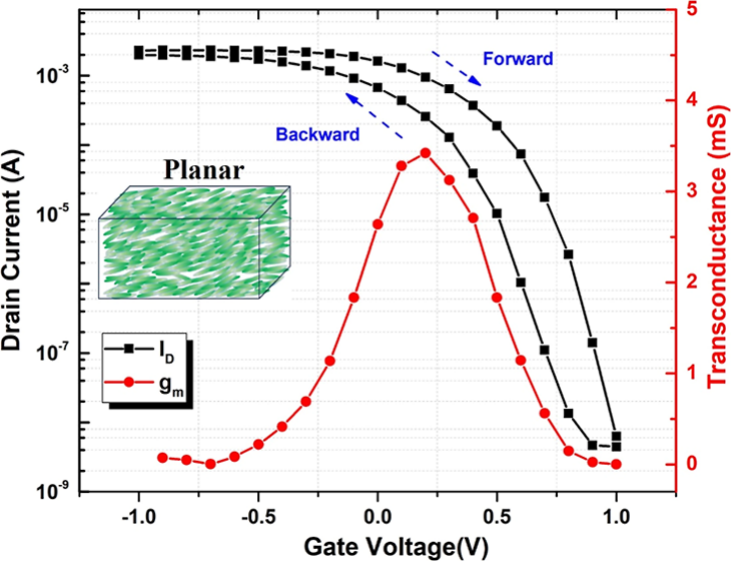

Organic electrochemical transistors (OECTs) have emerged as attractive devices for bioelectronics, wearable electronics, soft robotics, and energy storage devices. The electrolyte, being a fundamental component of OECTs, plays a crucial role in their performance. Recently, it has been demonstrated that ionic liquid crystal elastomers (iLCEs) can be used as a solid electrolyte for OECTs. Their capabilities, however, have only been shown for relatively large size substrate-free OECTs. Here, we study the influence of the different alignments of iLCEs on steady state and transient behavior of OECTs using a lateral geometry with source, drain, and gate in the same plane. We achieve excellent electrical response with an ON/OFF switching ratio of >10^5^ and minimal leakage current. The normalized maximum transconductance g_m_/w of the most sensitive iLCE was found to be 33 S m^–1^, which is one of the highest among all solid-state-based OECTs reported so far. Additionally, iLCEs show high stability and can be removed and reattached multiple times to the same OECT device without decreasing performance.

## 1 Introduction

The study of organic electrochemical transistors (OECTs) has been growing since their discovery in the mid-1980 s.^1^ It has become one of the most promising candidates for organic bioelectronic applications, as the device architecture enables simple electrical readout, convenient fabrication, and fast manufacturing on flexible substrates.^2−4^ OECTs exhibit a hybrid electrical-ionic conduction mechanism and thus enable significant electric current modulations under low gate voltages. OECTs enable detection of ions,^5^ metabolites,^6^ hormones,^7^ DNA,^8^ dopamine,^9^ and activity of electrically active cells or tissues.^10^

Depending on the way the charge carrier density is modulated, the OECTs operate in either depletion or accumulation mode. Most OECTs rely on the p-type organic semiconductor poly(3,4-ethylenedioxythiophene) polystyrenesulfonate (PEDOT:PSS). Other common materials include poly(3-carboxy-pentyl-thiphene) (P3CPT), poly(2-(3,3′-bis(2-(2-(2-methoxyethoxy) ethoxy) ethoxy)-[2,2′-bithiophen]-5-yl)thieno[3,2-*b*]thiophene) (p(g2T-TT)), and poly(3-hexylthiophene) (P3HT).^11^ Since PEDOT:PSS OECTs have been fabricated with various geometries and thicknesses to improve the performance of the OECTs, it has become the most heavily used channel material operating in depletion mode.

Besides the structural parameters (such as channel geometry, gate electrode type, and semiconductor materials), the electrolyte also significantly influences the behavior of the OECTs and their targeted use. The choice of the electrolyte and the concentration of ions within the electrolyte play a crucial role, particularly in determining the ON/OFF ratio of the OECTs. Most of the OECTs use a simple salt and water mixture as the electrolyte. In addition to gels compounds and polymers, room temperature ionic liquids, such as 1-ethyl-3-methylimidazolium ethyl sulfate (C2MIM EtSo_4_), which are as well used in supercapacitors or as electrolyte within solid hydrogels^12,13^ or ion-gels,^14^ can be used in OECTs. Solid-state electrolytes, including hydrogel based ionic conductive materials, are susceptible to moisture, weight loss, and dehydration leading to short operational lifetime.^15,16^ Additionally, solid electrolytes typically present lower ionic conductivity than liquid electrolytes and thus result in inefficient ion transport and slow switching response.^17^

Liquid crystal elastomers (LCEs)^18^ are lightly cross-linked polymers in which the repeating units are elongated liquid crystal (LC) molecules.^19,20^ The majority of LCs, especially those used in electro-optics and displays (LCDs), are practically insulators, and LCEs as well are usually highly insulating. In contrast, ionic LCs,^21^ in which ionic or polar groups,^22,23^ or conjugated moieties, are incorporated in the molecules for electron or hole transport,^21,24^ can be used in energy generation and storage applications.^25−27^

The iLCEs studied by our group consist of insulating LCEs, in which we dispersed 15–40 wt % of ionic liquids that phase separate from the LCE, forming continuous electrically conducting ion channels.^28,29^ In spite of that, the ionic liquids are not leaking out from the iLCE, thus representing solid electrolytes. Recently, Rajapaksa et al.^28^ demonstrated that ionic liquid crystal elastomers (iLCEs) can be used as efficient electrolytes in flexible, substrate-free OECTs with a performance that depends on the overall alignment of the liquid crystal director. Although the first results with iLCE-OECTs already showed promising results such as a maximum normalized transconductance of 7 S m^–1^, a switching time of around 2 s and an ON/OFF ratio of about 163, these parameters are far from optimized and need to be studied further.^28^

Here, we study the influence of different alignments of iLCEs films used as solid electrolyte on OECT operation. We observe that iLCEs adhere reliably to the device but can be peeled off easily without damaging the device, opening the possibility to exchange or renew the electrolyte. Moreover, we achieved excellent performances with switching ratio >10^5^ and normalized transconductance as large as 33 S m^–1^. Overall, iLCEs constitute a new class of solid electrolytes that provide high ON/OFF ratio and minimal gate leakage and remain stable without hydrolysis or weight loss.

## 2 Materials and Methods

The studied iLCE-OECTs are prepared on 4 inch silicon wafers coated by a 5 μm thick polyimide layer. The metal contacts (drain, source, and gate electrodes) are formed by sputtering 200 nm gold and structuring the gold film by standard processes.^30^ A second, 300 nm thick polyimide layer is deposited to act as a passivation layer for the contacts, followed by a third polyimide layer (5 μm) used as mechanical shadow mask for channel structuring. Both layers are also structured using standard photolithography and reactive ion etching to open the channels, gate, and contact pads. Details of the fabrication process were previously discussed by Barbosa et al.^30^

**Figure 1:**
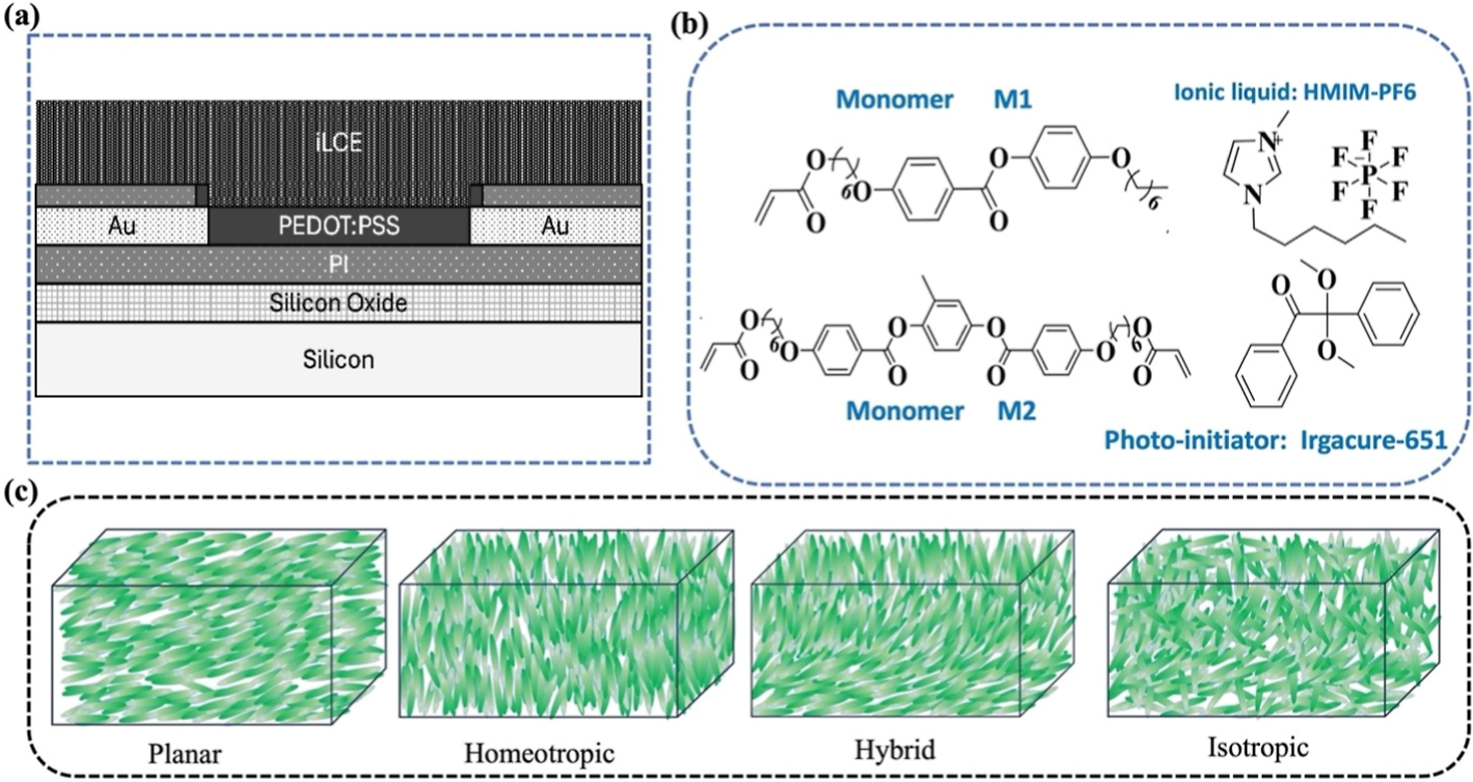
Materials and the schematic of the side view of fabricated OECT and illustration of the LC director structure in different configurations. (a) Schematic diagram of organic electrochemical transistors design: side view of the OECT, (b) molecular structures of compounds of iLCEs, and (c) illustration of the LC director structure in different configurations.

PEDOT:PSS films were formed by mixing 20 mL of PH 1000 solution from Clevios with 5 mL ethylene glycol (EG), 50 μL dodecyl benzenesulfonic acid (DBSA), and 242 μL of (3-glycidyloxypropyl) trimethoxysilance (GOPS) from Sigma-Aldrich. This solution was spin-coated over the substrates for 60 s at a speed of 1000 rpm and annealed at 110 °C for 10 min. The shadow mask layer of polyimide is then peeled off to reveal the PEDOT:PSS channels. In this design, the channel lengths were varied from 100 μm to 1 mm, and the channel widths were varied between 50 and 600 μm. There is a 10 μm overlap of the semiconductor layer with the source and drain electrode. A 3.5 × 3.5 mm gate electrode was also coated with PEDOT:PSS, which lies in plane with the channels. The schematic of the side view of the channel of these OECTs is shown in Figure 1a.

The gate electrodes are made of gold coated with PEDOT:PSS. Such PEDOT:PSS-coated gold gate electrodes have excellent gating capacity comparable to gating obtained by Ag/AgCl electrodes.^31^ Measurements have been done on 8 chips with different channel widths from 50 to 600 μm; each chip had 10 transistors with different channel lengths from 100 to 1000 μm. Although out of 80 transistors, 11 devices failed to show any modulation in drain current or showed unusually low on–off ratios, the majority showed regular behavior. The devices were prepared in the cleanroom of the Institute for Microsensors, Microactuators, and Microsystems (IMSAS), University of Bremen. The device design used here is shown in Figures S1 and S2 of the Supporting Information.

The solid electrolyte consists of LC monomers, cross-linker, and ionic liquid (see Figure 1b). As previously reported,^28,32,33^ the LCE precursor solution was prepared by mixing a monofunctional acrylate M1 (4-(6-Acryloxy-hex-1-yl-oxy) phenyl-4-(hexyloxy) benzoate) and a bifunctional acrylate M2 (1,4-Bis-[4-(6-acryloyloxyhexyloxy) benzoyloxy]-2-methylben-zene) with a photoinitiator in 87:12:1 weight ratio and heated to 80 °C. Then, 25 wt % ionic liquid (HMIM-PF_6_) was added and stirred for 10 min. There is no water or any solvent to form the elastomer. In our experiments, the mixture was filled in 200 μm thick cells between 1 mm thick borosilicate glass substrates, but any other thickness can be used by adjusting the cell gap. The LC mixture was lightly cross-linked by a Black-Ray, Model B-100AP/R 5 mW/cm^2^ intensity UV light at 365 nm wavelength either in the isotropic phase at 80 °C or in 53–55 °C temperature range in the nematic phase with different alignments (planar, homeotropic, and hybrid) for 10 min. The director structures of the four different iLCEs prepared using the surface alignment method are shown in Figure 1c. The planar alignment is parallel to the surface substrate, and homeotropic is perpendicular to the surface, whereas hybrid combines both planar and homeotropic. The isotropic structure is made in the isotropic phase, and at room temperature, it is also in the nematic phase containing randomly aligned sub micrometer size domains.

Planar (PI-2555) and homeotropic (PI-5661) alignment layers were spin-coated on the substrates in the clean room of the Advanced Materials and Liquid Crystal Institute of Kent State University. After deposition, they were soft backed for 5 min at 80 °C. PI-2555 was hard baked for 1 h at 200 °C and PI-5661 was hard baked 1 h at 160 °C for curing. Finally, the substrates coated with PI-2555 were rubbed unidirectionally to align the liquid crystal director in the plane parallel to the rubbing direction.

## 3 Results and Discussion

**Figure 2:**
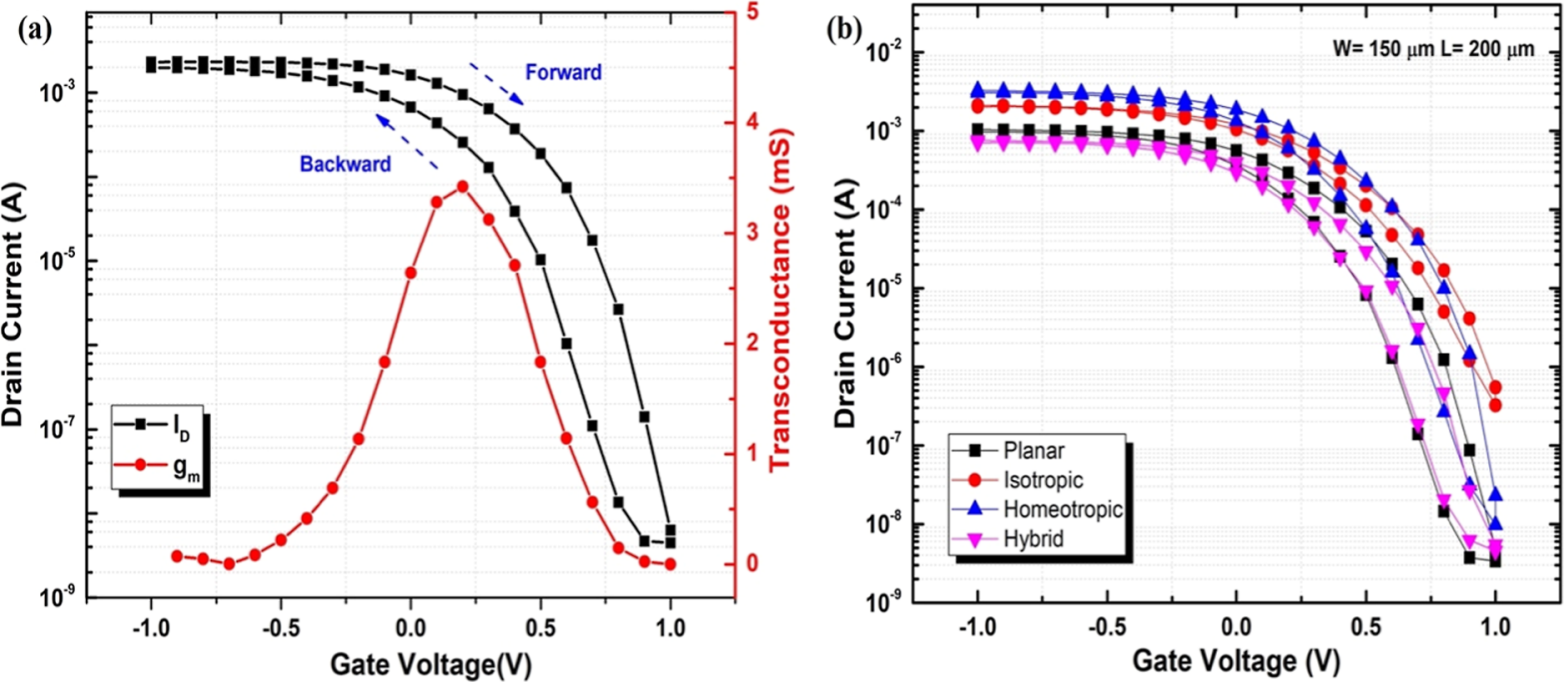
(a) Transfer characteristic (drain current *I*_D_ versus gate voltage *V*_G_ between −1 and 1 V, solid black squares) of iLCE-OECTs at 200 μm channel length and 150 μm channel width at a drain bias of *V*_D_ = −0.5 V, and the corresponding transconductance peak (solid red circle); (b) typical transfer curves of iLCE-OECTs with different LC director alignments.

The transfer (drain current *I*_D_ versus gate voltage *V*_G_) and transconductance g_m_ (the change in drain current with respect to gate voltage ) curves for a planar iLCE electrolyte with 200 μm channel length and 150 μm channel width at drain bias of *V*_D_ = −0.5 *V* are shown in Figure 2a. This sample shows some hysteresis and a peak transconductance *g*_m_ ∼ 3.5 mS, which is comparable with the results obtained in aqueous NaCl and other ionic liquid electrolytes.^34^

Figure 2b compares the transfer curves measured at a drain voltage of *V*_D_ = −0.5 for different LC director orientations. It can be seen that LC director alignment has a profound effect on the transistor performance. The ON/OFF ratio for planar, homeotropic, and hybrid alignments is ∼10^6^, while for the isotropic structure, it is only ∼10^4^. This emphasizes the importance of the alignment that facilitates the movement of cations resulting in higher ionic conductivity and faster doping/dedoping of the bulk semiconductor.^14^

**Figure 3:**
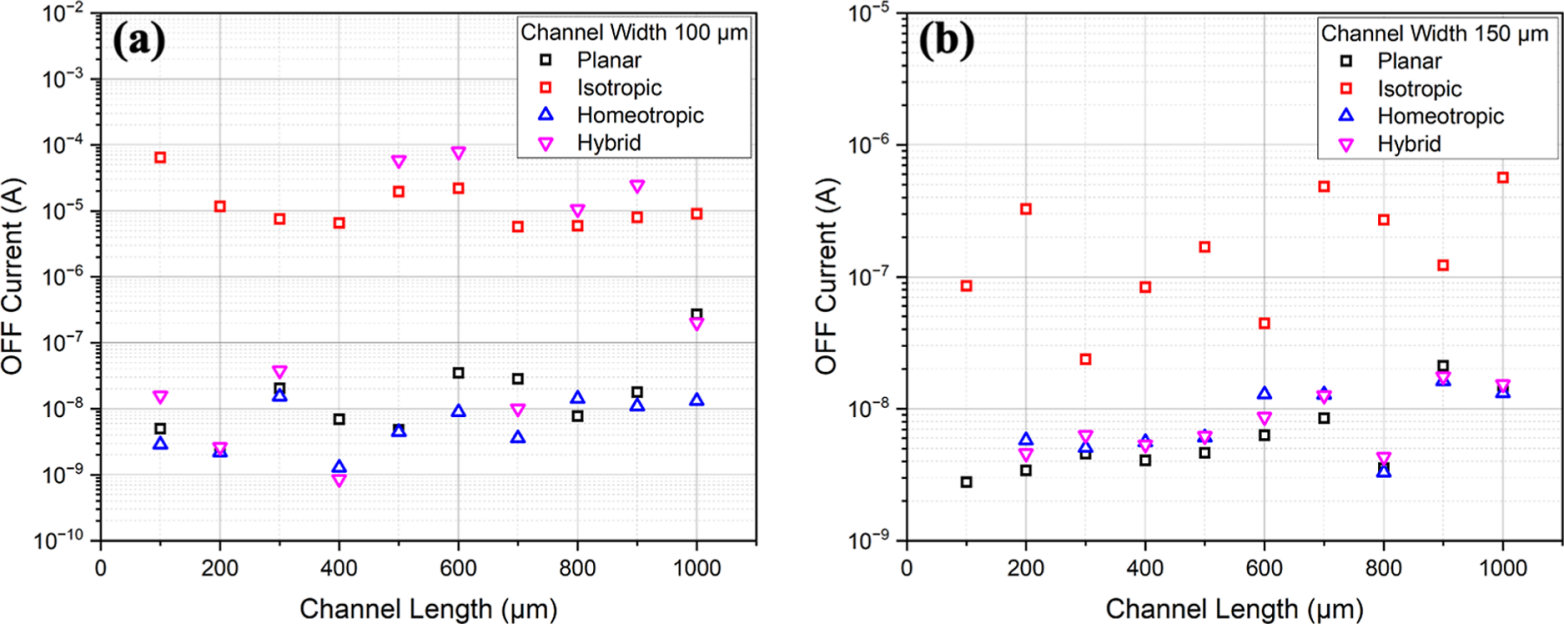
OFF current as a function of channel length for different LC alignments. (a) Channel width is 100 μm; (b) channel width is 150 μm.

This increase in ON/OFF ratio is caused by differences in the OFF current with changing alignments, as shown in Figure 3. Films that were crossed-linked in the nematic phase with planar, homeotropic, or hybrid alignments have 2 to 3 orders of magnitude smaller OFF currents than the films cross-linked in their isotropic phase.

According to prior studies,^28,29,33^ the actuation performances and flexoionic performances correlated to the conductivities that in the homeotropic and hybrid alignments are considerably smaller than in the planar and isotropic samples. Polarizing optical microscopy measurements showed that these are due to the presence of a defect wall in between the planar and homeotropic alignment in the hybrid cells and due to the smaller ion mobility for the homeotropic alignment that suppresses ion motion considerably. These trends are not reflected in the ON/Off ratios in the transistors that are the smallest in samples that were cross-linked in the isotropic phase, i.e., they do not have any defects that would trap the ions. This leads to an increase in the current in the OFF state, thereby reducing the ON/OFF ratio.

Moreover, the samples with hybrid alignment showed less reproducible OFF currents compared to the other films in the nematic phase, which can be explained by a defect wall between the planar and homeotropic sides dictated by the different surface alignments. Both, planar and homeotropic sides in contact with the PEDOT:PSS layer was tested but did not show a significant difference within the measurement error.

**Figure 4:**
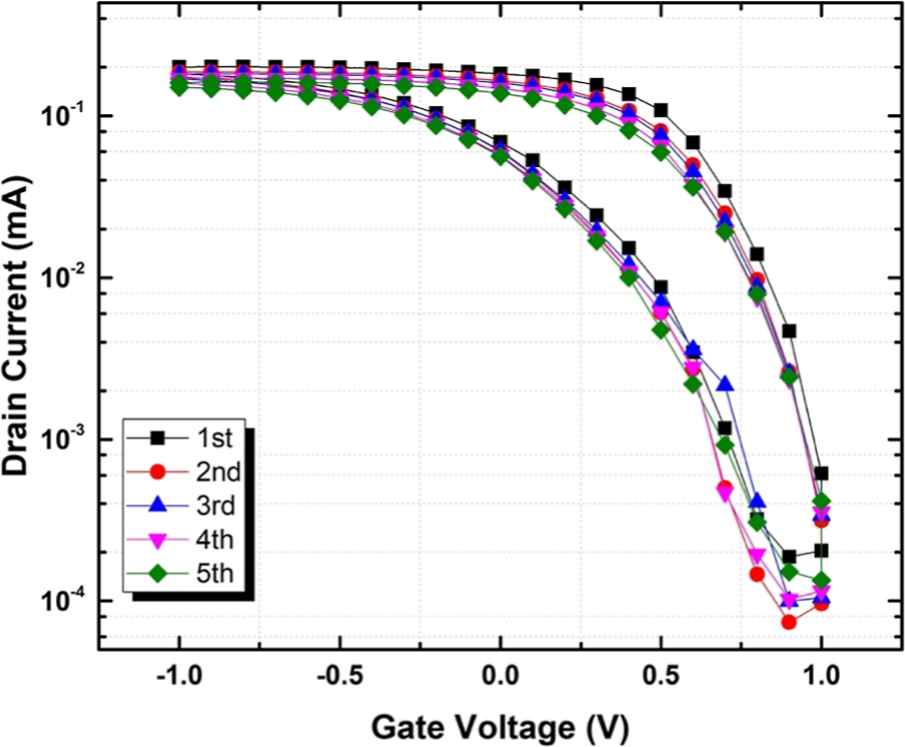
Transfer characteristic of the same OECT with peeling the same iLCE film and reattached several times.

These measurements were done on the same OECT devices but with replacement of the solid electrolyte (iLCEs) by peeling off the measured one and replacing it with a new iLCE. Importantly, this could be done multiple times without any leakage or damage to the OECT surface. Figure 4 compares several cycles of peeling and reattaching the same film on the same OECT measured in ambient air. The hysteresis does not show any change, and no degradation in drain current was observed.

The iLCEs stability has been reported for the same iLCEs as a flexible free substrate in OECTs.^35^ The device showed slight degradation with the drain current after 100 cycles. This gradual decrease was due to the PEDOT: PSS layer. Moreover, since the material is solid (rubbery) after cross-linking, no milking of the ionic liquid was observed in the experiments nor any effect related to humidity.

As can be seen in Figures 2 and 4, there is a hysteresis between the current measured for increasing and decreasing voltages; i.e., the currents during the forward sweep are slightly larger than the ones during the backward sweep.

**Figure 5:**
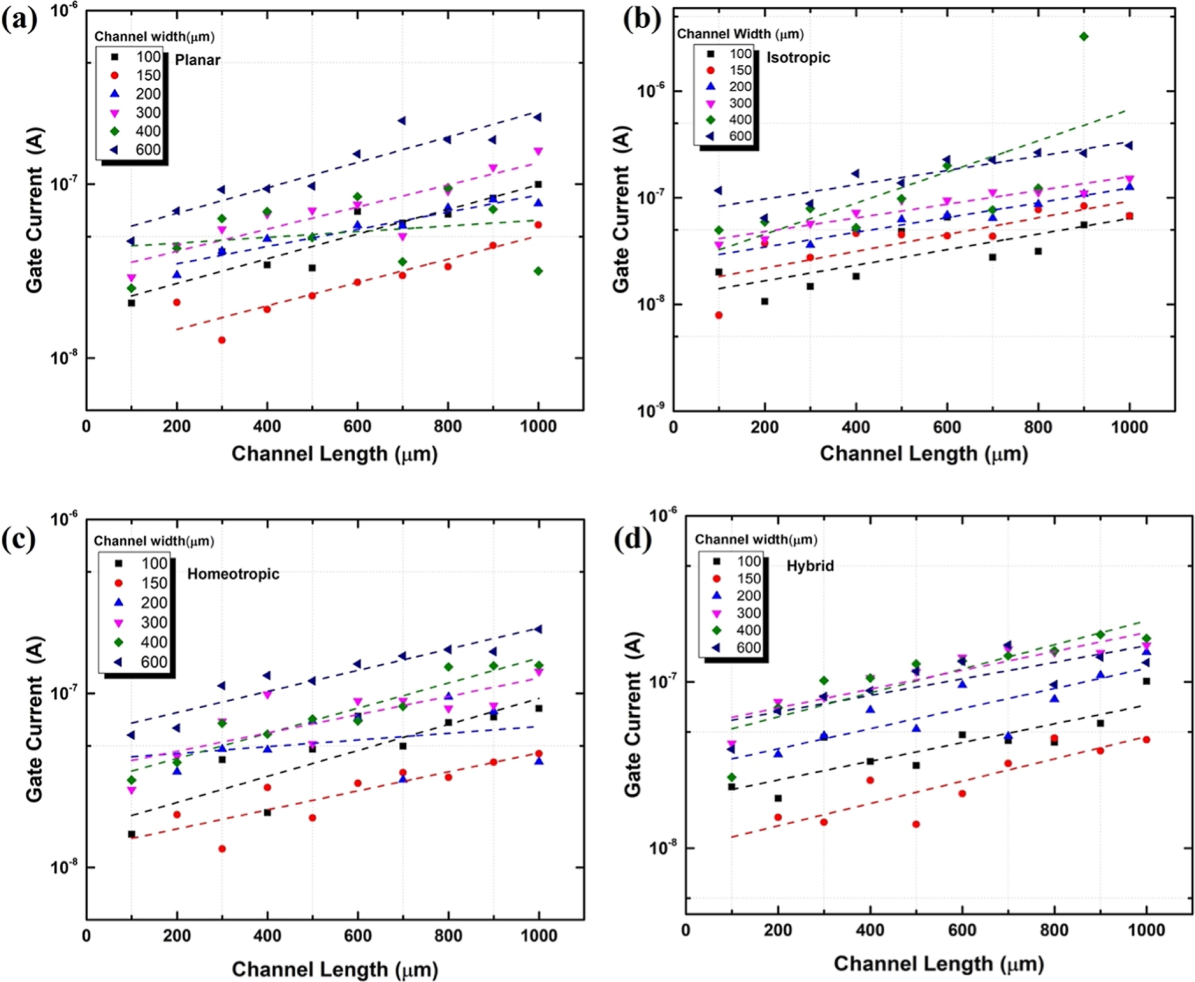
Gate current at (1 V) as a function channel length for channel widths between 100 and 600 μm. (a) Planar, (b) isotropic, (c) homeotropic, and (d) hybrid LC director structures.

**Figure 6:**
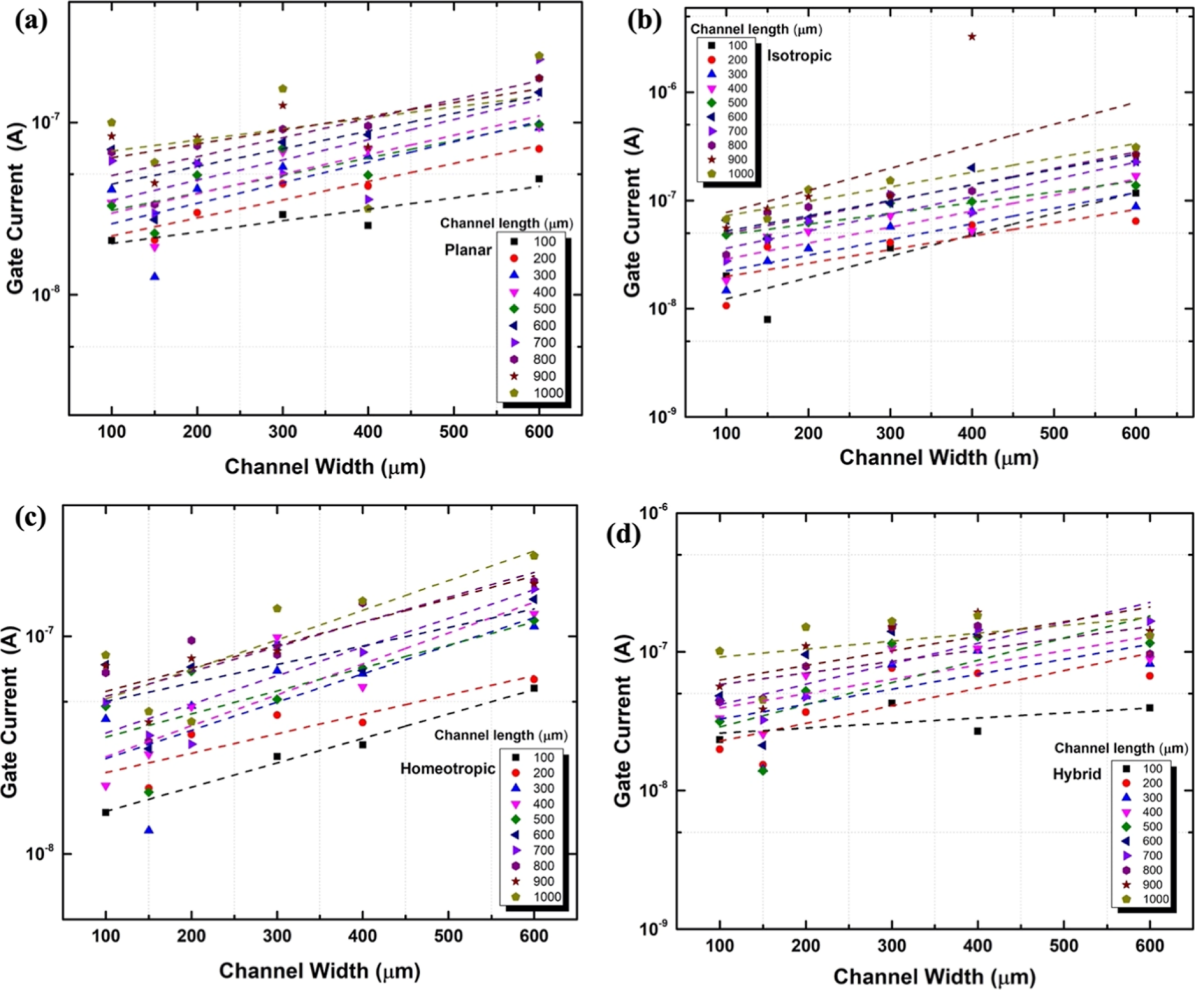
Gate current at (1 V) as a function of the channel width for channel lengths between 100 and 1000 μm for different alignments. (a) Planar, (b) isotropic, (c) homeotropic, and (d) hybrid LC director structures.

Figures 5 and 6 show the effect of the channel length (Figure 5) and width (Figure 6) on the gate-current for the four different alignments [(a): planar, (b): isotropic, (c): homeotropic, and (d): hybrid].

Within the error of the measurements, linear dependencies are found between the gate current and the channel length for all channel widths. Both Figures 5 and 6 show that the peak gate current scales linearly with both the channel length and the channel width, i.e., with the channel area. Moreover, there is no significant difference in gate current with changing alignment, and for all alignments shown in Figures 5 and 6, the gate current is below 1 μA.

At the saturation region, the relationship between *g*_m_ and *W*/*L* can be expressed as^36^
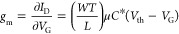
1Here, μ is the electronic charge carrier mobility, C* is the volumetric capacitance (capacitance per volume), *V*_th_ is the threshold voltage, *V*_G_ is the gate voltage at the high *g*_m_, and *W*, *T* = 100 nm, and *L* are the width, thickness, and length of the channel, respectively. This equation shows that the maximum transconductance scales linearly with the channel length and thickness regardless of the materials and device configuration.

**Figure 7:**
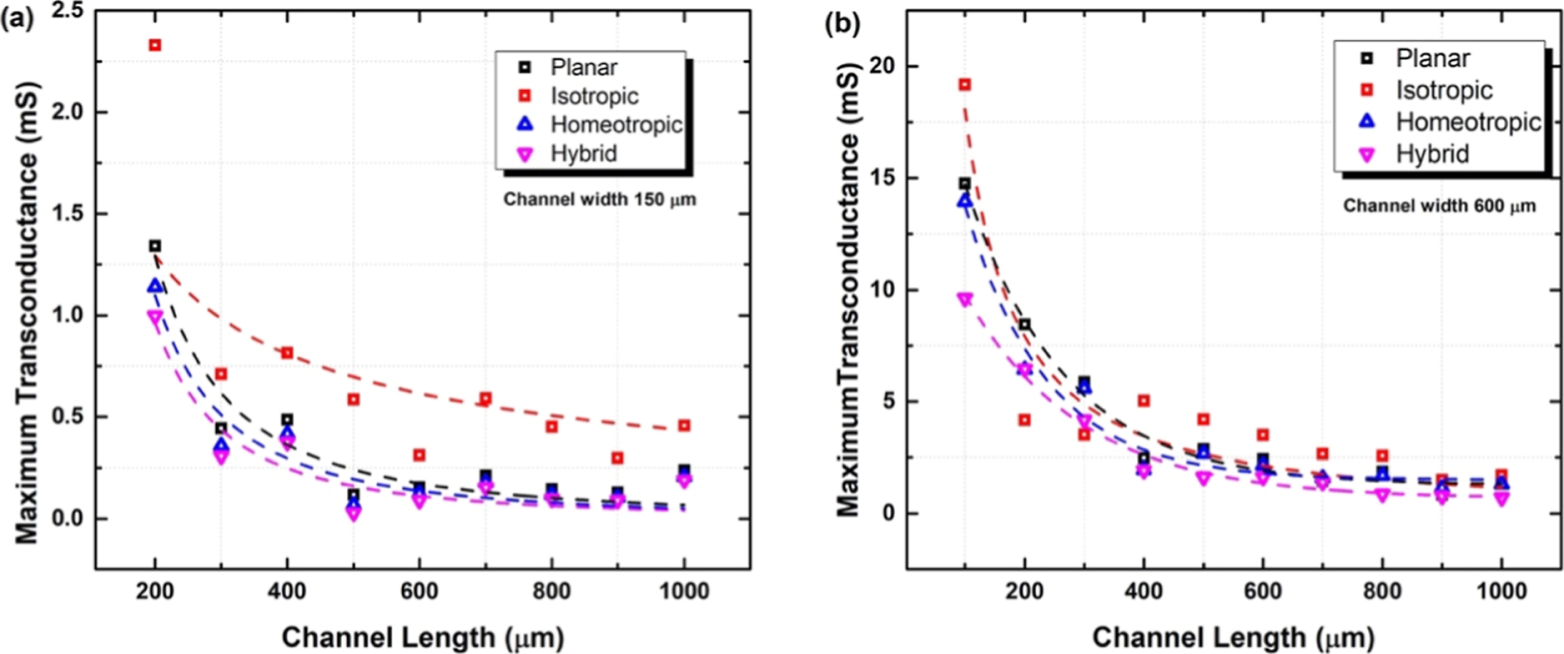
Maximum transconductance as a function of channel length for different LC alignments. (a) Channel width is 150 μm; (b) channel width is 600 μm.

Figure 7 plots the maximum transconductance *g*_m_ of the OECTs as a function of channel length for transistors with 150 and 600 μm as channel width. For both channel widths, the transconductance drops with the inverse of the channel length, which is in line with the results in eq 1.

Comparing Figure 7a,b, it is seen as well that increasing the channel width from 100 to 600 μm results in a higher drain current. Overall, it can be seen that the transconductance is proportional to the ratio of channel width to length , which is again in line with eq 1.

Figure 7 shows as well that the electrolyte with isotropic alignment results in higher *g*_m_ values compared with the other alignments. The highest transconductance of *g*_m_ ∼ 20 mS is found for a device with *L* = 100 μm and *W* = 600 μm. For the same device, the normalized transconductance *g*_m_/*W* values are 33, 25, 24.5, and 17 S m^–1^ for isotropic, planar, homeotropic, and hybrid alignments. This trend is identical to the results reported by us previously in^28^ only with lower normalized transconductance with 7, 5.5, 2.5, and 1 S m^–1^. Overall, due to the volumetric characteristic of the PEDOT:PSS layer, most of the OECTs showed higher transconductance (>4 mS). The combination of the large transconductance and low voltage operation is advantageous, making solid-state OECTs ideal for electron transducers.

**Figure 8:**
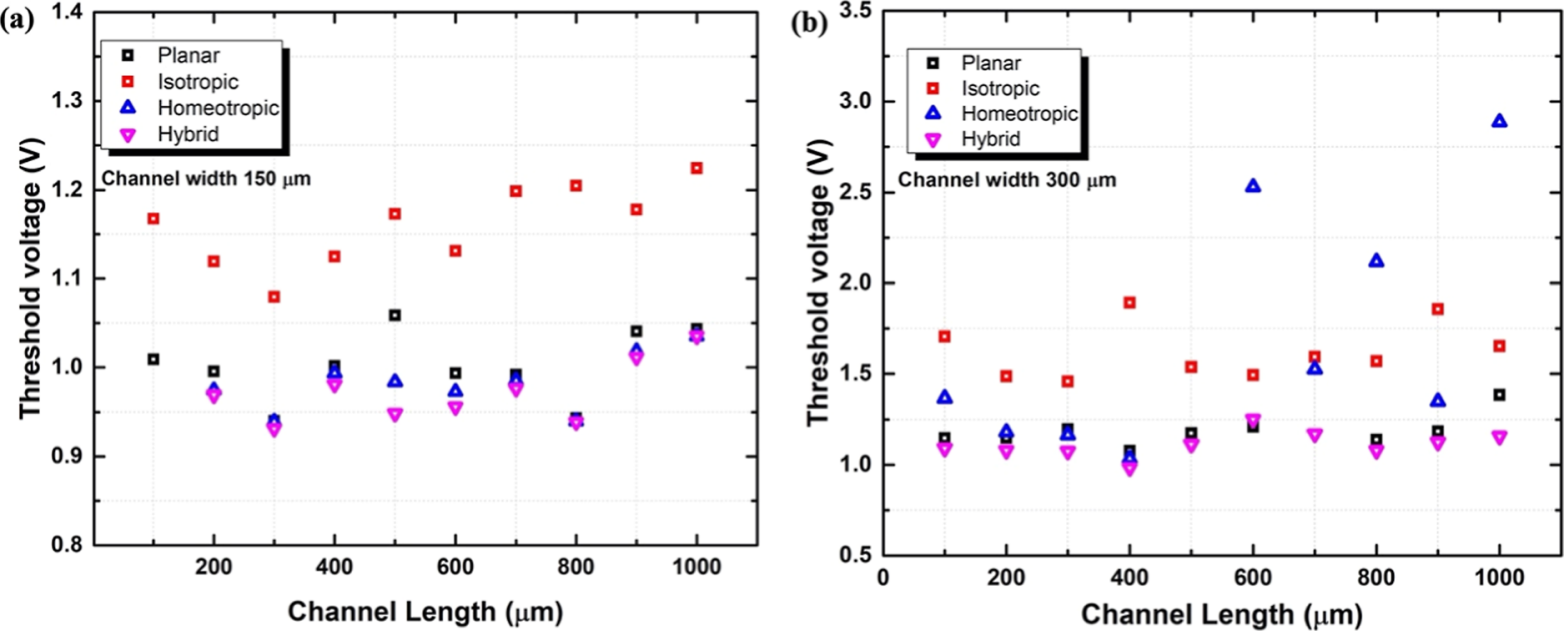
Threshold voltage extraction by linear extrapolation vs the different channel length for different alignments (a) channel width is 150 μm; (b) channel width is 300 μm.

The detailed OECT performance of threshold voltage (*V*_th_) at various channel geometry is summarized in Figure 8. Here, *V*_th_ was obtained from linear extrapolation from a plot of the square root of the drain current.^37^ For iLCEs with different alignments, the threshold voltage *V*_th_ was in the range of 0.9–2.8 V. There is no difference between samples with different alignments. There are a few outliers, which might be caused by imperfect dedoping of the active channel.

**Figure 9:**
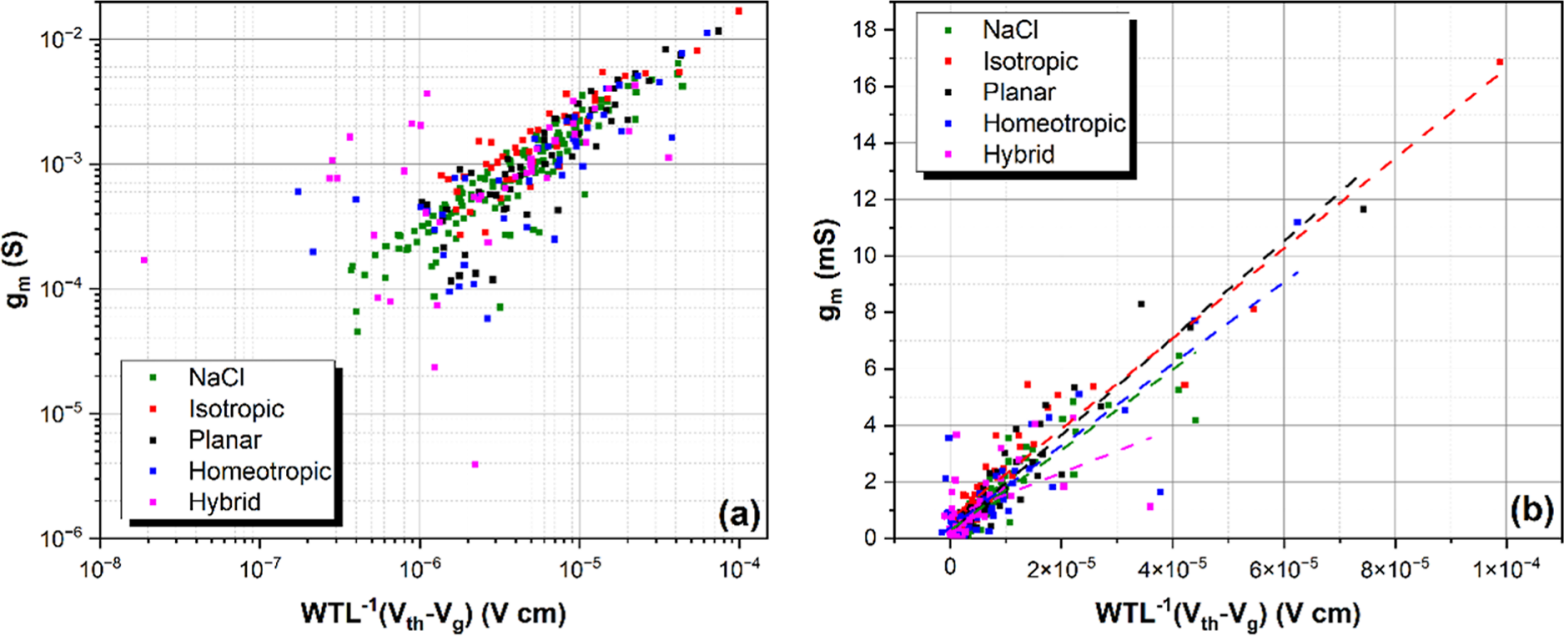
Maximum transconductance of all iLCE-OECTs in comparison to an OECT-containing NaCl electrolyte as a function of the channel geometry, *WT*/*L*. (a) Log–Log scale; (b) linear scale.

Figure 9 compares the maximum transconductances as a function of the channel geometry, *WT*/*L* of all iLCE-OECTs with an OECT containing a NaCl electrolyte. Within the measurement errors, the maximum transconductances of the iLCEs films with different alignments are proportional to *WT*/*L*. Furthermore, the proportionality of the transconductance on the geometric factor *WT*/*L* shows that the μC* product is constant across a wide geometrical range.

According to eq 1, based on Bernards and Malliaras model,^3^ the slope of the plot of maximum transconductance *g*_m_ vs  is proportional to μC*.^38^ This product is important to extract since it provides information about the performance of the particular combination of electrolyte and channel material. The mobility of PEDOT:PSS was already reported in the literature^38,39^ with 1.9 ± 1.3 cm^2^ V^–1^ s^–1^. According to previous results,^40^ this mobility might vary with ion concentration and/or gate voltage. However, since we intend to compare the influence of alignment of the iLCE on the transistor’s performance, the voltage dependence of the mobility was not studied further here.

Furthermore, Figure 9 shows scattering of the measured maximum transconductance, which may be attributed to measurement error, nonuniformities in film formation, edge effects, and their amplified effects in small devices.^38^

For comparison, we tested 100 mM NaCl as liquid electrolyte on the same OECT geometry. The behavior of the iLCE-OECT and NaCl-OECT devices are similar, showing linear dependence with slightly saturation for . This might be the result of parasitic series resistance which are caused by imperfect injection or extraction at the source and drain contacts.^40^ Here, μC* was extracted by linear extrapolation of the slope of maximum transconductance *g*_m_ vs  (see Figure 9). For fitting, samples with unphysically large threshold voltages (here: larger than 2 V) were excluded. With these constraints, the fittings resulted in μC* values of 142.3 ± 5.52, 159, 61 ± 7, 171.5 ± 8.2, 144.7 ± 11.9, and 77.3 ± 20 *F*/(cm·*V*·s) for NaCl, isotropic, planar, homeotropic, and hybrid cells. This means that the performance of devices with differently aligned elastomers is close except for the hybrid sample, which shows consistently lower performance.

Note that the measured slope of the NaCl-OECT is higher than 47 F cm^–1^ V^–1^ s^–1^ that was reported in the literature.^38^ Furthermore, all the μC* values are in the range that was reported for p-type OECTs using liquid electrolytes (1.7–522 F cm^–1^ V^–1^ s^–1^).^11^ We also note that the particular method of extracting the threshold voltage has a large effect on the resulting μC* values. Using a linear fit of the square root of the drain current (the method outlined in^41^), larger μC* products are obtained from identical data.

Clearly, a universally accepted method to extract the threshold voltage for an OECT is missing, and the concept and meaning of the threshold voltage for an OECTs is still not well understood. However, there are a number of factors that influence the μC* values, such as the nature of the electrolyte, the size, charge, and polarity of ions that have influence on ion penetration and their mobilities. For example, in a solid-state film, the large ion may not be able to access the electroactive volume, which may limit C*.^38^ Moreover, Shahi et al.^42^ reported that the μC* product is being greater when extracted from the *g*_m_ in OECTs compared to when the μC* measured independently. The reason for this is not clear either because *g*_m_ is voltage-dependent or μC* is maximum at peak *g*_m_.

**Figure 10:**
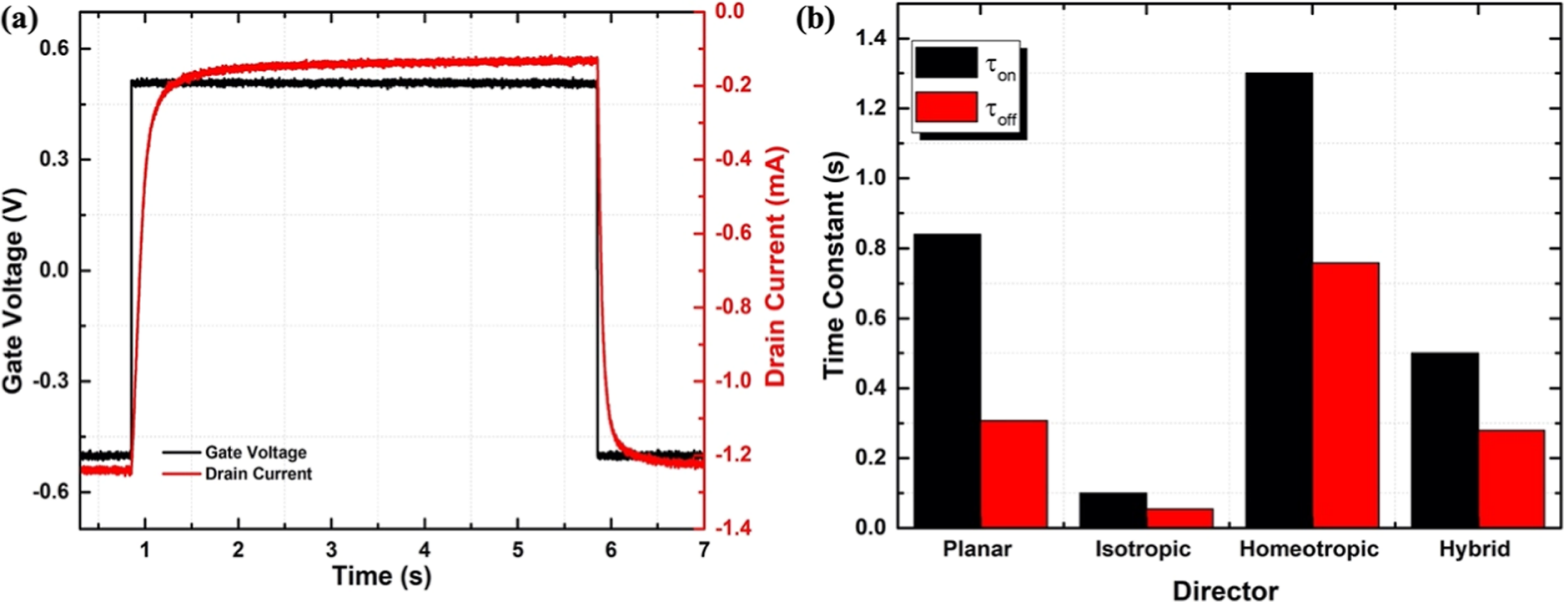
Summary of the response time measurements for an OECT with a channel length *L* = 100 μm, channel width *W* = 150 μm, and thickness of *T* = 100 nm. (a) Drain current (red data points) in response to square wave gate signal (black data points) at *V*_D_ = −0.5 *V*. (b) Switching in response to from negative to positive and positive to negative gate voltages for different alignments.

Nevertheless, the μC* value governs only the steady state characteristics of an OECT. We also investigated the response times of all of the studied iLCE-OECTs. Figure 10 summarizes the response time for an OECT with a channel length *L* = 100 μm, channel width *W* = 150 μm, and thickness *T* = 100 nm. Figure 10a shows the response to a *V*_G_ = ± 0.5 V square-wave voltage at 0.1 Hz applied to the gate electrode, while the drain voltage kept at *V*_D_ = −0.5 V. The characteristic response times of the drain current during the on and off switching are τ_on_ ∼ 100 ms and τ_off_ ∼ 54.2 ms, respectively. These are an order of magnitude smaller than what was reported previously in free-standing iLCE-OECTs with a much larger area.^28^Figure 10b compares the response times measured for differently aligned iLCEs. We found that the isotropic structure shows the fastest response. This likely related to the lack of defects when the cross-linker formed in the isotropic phase, while the defects are present, when the iLCEs are cross-linked in the nematic phase.^43−45^

Additionally, the switching time strongly depends on the properties of the electrolyte, where a higher conductivity gives faster switching. The isotropic and planar alignments have higher conductivity compared to the other alignments.^28^ Furthermore, it can also be influenced by many factors such as the concentration of the electrolyte. Moreover, using LCE with the nanosegregated structure will lead to fast doping and dedoping of the channel since it has ion-pathways,^23^ and this will achieve faster switching time.

## 4 Conclusions

With the aim of achieving high performance OECTs using iLCEs as solid electrolytes, in this work, we have investigated iLCE-OECTs with channel lengths varying from 100 to 1000 μm and channel width between 50 and 600 μm. We studied the effects of the iLCE alignments on the OECT performance. We found higher switching ratios (>10^5^) and higher normalized transconductances (33 S m^–1^) comparable to other OECTs with solid electrolytes. The different alignments of the LCE have a large effect on the performance of the OECTs. The iLCEs that were crossed-linked in the nematic phase showed a higher ON/OFF ratio due to the lower OFF current compared to the film with the isotropic phase. Our elastomer showed stability and reusability, and it can be used several times on the same OECT before it physically gets damaged. Moreover, the reproducibility of the device showed no degradation in the drain current when we peel and place the elastomer. Finally, the same iLCE films have been studied as bending sensors^35^ and as actuators^33^ where their durability was checked well above 100 cycles.

The electrolyte with isotropic alignment shows faster transient response with τ_on_ ∼ 100 ms and τ_off_ ∼ 54.2 ms, whereas the other alignments show a slower response, which might be due to trap generation by defects in the LCE. Importantly, as a result of the rubbery nature of the iLCEs, they can be peeled off and reattached to the OECT multiple times without affecting the performance of the devices. Therefore, iLCE-OECTs represent a new approach in the design of materials for OECTs and will contribute to the development of OECTs. These results not only verify the importance of the presented results but also imply the need for further studies toward a better understanding of the underlying mechanisms of switching and stability of iLCE-OECTs as necessary steps toward various wearable applications such as sensing and biomedical applications.

Key performance indicators of this work are summarized in Table 1.

**Table 1:** Comparison of the Performances of iLCEs With Different Alignments as Solid Electrolyte of OECT

iLCEs alignments (electrolyte)	channel material	*V*_t*h*_ (V)	[μC*]^a^ (F cm^–1^ *V*^–1^ s^–1^)	OFF current (A)	*I*ON/*I*OF
isotropic	PEDOT:PSS	1.12	159.61 ± 7	10^–6^	10^4^
planar	PEDOT:PSS	1	171.5 ± 8.2	10^–8^	∼10^6^
homeotropic	PEDOT:PSS	1	144.7 ± 11.9	10^–8^	∼10^6^
hybrid	PEDOT:PSS	1	77.3 ± 20	10^–8^	∼10^6^

a Calculated from the slope of *g*_m_ as a function of .

## Supporting Information Available

Mask layout of the Si chip before and after deposition, an image of the ionic liquid crystal elastomer as a solid electrolyte on top of the OECT, and the molecular structures of substances used to make iLCE films (PDF)

## References

[ref1] White H. S.; Kittlesen G. P.; Wrighton M. S. Chemical Derivatization of an Array of Three Gold with Polypyrrole: Fabrication of a Molecule-Based Transistor. J. Am. Chem. Soc. 1984, 106 (18), 5375–5377. 10.1021/ja00330a070.

[ref2] Khodagholy D.; Gurfinkel M.; Stavrinidou E.; Leleux P.; Herve T.; Sanaur S.; Malliaras G. G. High Speed and High Density Organic Electrochemical Transistor Arrays. Appl. Phys. Lett. 2011, 99 (16), 163304 10.1063/1.3652912.

[ref3] Bernards D. A.; Malliaras G. G. Steady-State and Transient Behavior of Organic Electrochemical Transistors. Adv. Funct. Mater. 2007, 17 (17), 3538–3544. 10.1002/adfm.200601239.

[ref4] Rivnay J.; Inal S.; Salleo A.; Owens R. M.; Berggren M.; Malliaras G. G. Organic Electrochemical Transistors. Nat. Rev. Mater. 2018, 3, 17086 10.1038/natrevmats.2017.86.

[ref5] Lin P.; Yan F.; Chan H. L. W. Ion-Sensitive Properties of Organic Electrochemical Transistors. ACS Appl. Mater. Interfaces 2010, 2 (6), 1637–1641. 10.1021/am100154e. 20499881

[ref6] Liao C.; Mak C.; Zhang M.; Chan H. L. W.; Yan F. Flexible Organic Electrochemical Transistors for Highly Selective Enzyme Biosensors and Used for Saliva Testing. Adv. Mater. 2015, 27 (4), 676–681. 10.1002/adma.201404378. 25469658

[ref7] Coppedè N.; Tarabella G.; Villani M.; Calestani D.; Iannotta S.; Zappettini A. Human Stress Monitoring through an Organic Cotton-Fiber Biosensor. J. Mater. Chem. B 2014, 2 (34), 5620–5626. 10.1039/C4TB00317A. 32262196

[ref8] Lin P.; Luo X.; Hsing I. M.; Yan F. Organic Electrochemical Transistors Integrated in Flexible Microfluidic Systems and Used for Label-Free DNA Sensing. Adv. Mater. 2011, 23 (35), 4035–4040. 10.1002/adma.201102017. 21793055

[ref9] Liao C.; Zhang M.; Niu L.; Zheng Z.; Yan F. Organic Electrochemical Transistors with Graphene-Modified Gate Electrodes for Highly Sensitive and Selective Dopamine Sensors. J. Mater. Chem. B 2014, 2 (2), 191–200. 10.1039/C3TB21079K. 32261606

[ref10] Campana A.; Cramer T.; Simon D. T.; Berggren M.; Biscarini F. Electrocardiographic Recording with Conformable Organic Electrochemical Transistor Fabricated on Resorbable Bioscaffold. Adv. Mater. 2014, 26 (23), 3874–3878. 10.1002/adma.201400263. 24644020

[ref11] Paudel P. R.; Tropp J.; Kaphle V.; Azoulay J. D.; Lüssem B. Organic Electrochemical Transistors - From Device Models to a Targeted Design of Materials. J. Mater. Chem. C 2021, 9 (31), 9761–9790. 10.1039/D1TC01601F.

[ref12] Cunha I.; Ferreira S. H.; Martins J.; Fortunato E.; Gaspar D.; Martins R.; Pereira L. Foldable and Recyclable Iontronic Cellulose Nanopaper for Low-Power Paper Electronics. Adv. Sustainable Syst. 2022, 6, 2200177 10.1002/adsu.202200177.

[ref13] Carvalho J. T.; Cunha I.; Coelho J.; Fortunato E.; Martins R.; Pereira L. Carbon-Yarn-Based Supercapacitors with in Situ Regenerated Cellulose Hydrogel for Sustainable Wearable Electronics. ACS Appl. Energy Mater. 2022, 5 (10), 11987–11996. 10.1021/acsaem.2c01222. 36311466 PMC9597547

[ref14] Kaphle V.; Liu S.; Keum C. M.; Lüssem B. Organic Electrochemical Transistors Based on Room Temperature Ionic Liquids: Performance and Stability. Phys. Status Solidi A 2018, 215 (24), 1800631 10.1002/pssa.201800631.

[ref15] Yuk H.; Lu B.; Zhao X. Hydrogel Bioelectronics. Chem. Soc. Rev. 2019, 48 (6), 1642–1667. 10.1039/C8CS00595H. 30474663

[ref16] Park D. H.; Park H. W.; Chung J. W.; Nam K.; Choi S.; Chung Y. S.; Hwang H.; Kim B. S.; Kim D. H. Highly Stretchable, High-Mobility, Free-Standing All-Organic Transistors Modulated by Solid-State Elastomer Electrolytes. Adv. Funct. Mater. 2019, 29 (18), 1808909 10.1002/adfm.201808909.

[ref17] Chen S.; Surendran A.; Wu X.; Leong W. L. Contact Modulated Ionic Transfer Doping in All-Solid-State Organic Electrochemical Transistor for Ultra-High Sensitive Tactile Perception at Low Operating Voltage. Adv. Funct. Mater. 2020, 30 (51), 2006186 10.1002/adfm.202006186.

[ref18] Warner M.; Terentjev E. M. Liquid Crystal Elastomers, 1st ed.; Clarendon Press: Oxford, 2006.

[ref19] de Gennes P.-G.; Prost J.; Pelcovits R. The Physics of Liquid Crystals. Phys. Today 1995, 48 (5), 70–71. 10.1063/1.2808028.

[ref20] de Gennes P. G.; Prost J. The Physics of Liquid Crystals, 2nd ed.; Claredon Press: Oxford, 1993.

[ref21] Kato T.; Yoshio M.; Ichikawa T.; Soberats B.; Ohno H.; Funahashi M. Transport of Ions and Electrons in Nanostructured Liquid Crystals. Nat. Rev. Mater. 2017, 2 (4), 17001 10.1038/natrevmats.2017.1.

[ref22] Kato T.; Mizoshita N.; Kishimoto K. Functional Liquid-Crystalline Assemblies: Self-Organized Soft Materials. Angew. Chem., Int. Ed. 2006, 45 (1), 38–68. 10.1002/anie.200501384. 16353263

[ref23] Kato T. From Nanostructured Liquid Crystals to Polymer-Based Electrolytes. Angew. Chem., Int. Ed. 2010, 49 (43), 7847–7848. 10.1002/anie.201000707. 20677293

[ref24] Adam D.; Closs F.; Frey T.; Funhoff D.; Haarer D.; Schuhmacher P.; Siemensmeyer K.; Siemensmeyer K. Transient Photoconductivity in a Discotic Liquid Crystal. Phys. Rev. Lett. 1993, 70 (4), 457–460. 10.1103/physrevlett.70.457. 10054117

[ref25] Yamanaka N.; Kawano R.; Kubo W.; Kitamura T.; Wada Y.; Watanabe M.; Yanagida S. Ionic Liquid Crystal as a Hole Transport Layer of Dye-Sensitized Solar Cells. Chem. Commun. 2005, (6), 740–742. 10.1039/b417610c. 15685322

[ref26] Kerr R. L.; Edwards J. P.; Jones S. C.; Elliott B. J.; Gin D. L. Effect of Varying the Composition and Nanostructure of Organic Carbonate-Containing Lyotropic Liquid Crystal Polymer Electrolytes on Their Ionic Conductivity. Polym. J. 2016, 48 (5), 635–643. 10.1038/pj.2015.119.

[ref27] Sakuda J.; Hosono E.; Yoshio M.; Ichikawa T.; Matsumoto T.; Ohno H.; Zhou H.; Kato T. Liquid-Crystalline Electrolytes for Lithium-Ion Batteries: Ordered Assemblies of a Mesogen-Containing Carbonate and a Lithium Salt. Adv. Funct. Mater. 2015, 25 (8), 1206–1212. 10.1002/adfm.201402509.

[ref28] Hemantha Rajapaksha C. P.; Paudel P. R.; Kodikara P. M. S. G.; Dahal D.; Dassanayake T. M.; Kaphle V.; Lüssem B.; Jákli A. Ionic Liquid Crystal Elastomers-Based Flexible Organic Electrochemical Transistors: Effect of Director Alignment of the Solid Electrolyte. Appl. Phys. Rev. 2022, 9 (1), 011415 10.1063/5.0077027.

[ref29] Rajapaksha C. P. H.; Gunathilaka M. D. T.; Narute S.; Albehaijan H.; Piedrahita C.; Paudel P.; Feng C.; Lüssem B.; Kyu T.; Jákli A. Flexo-Ionic Effect of Ionic Liquid Crystal Elastomers. Molecules 2021, 26 (14), 4234 10.3390/molecules26144234. 34299509 PMC8304522

[ref30] Barbosa H. F.; Asyuda A.; Skowrons M.; Schander A.; Lüssem B.; Lüssem B. Processing of Organic Electrochemical Transistors. MRS Commun. 2024, 14 (2), 132–148. 10.1557/s43579-024-00521-y.

[ref31] Koutsouras D. A.; Torricelli F.; Gkoupidenis P.; Blom P. W. M. Efficient Gating of Organic Electrochemical Transistors with In-Plane Gate Electrodes. Adv. Mater. Technol. 2021, 6 (12), 2100732 10.1002/admt.202100732.

[ref32] Alyami A.; Rajapaksha C. P. H.; Feng C.; Paudel P. R.; Paul A.; Adaka A.; Dharmarathna R.; Lüssem B.; Jákli A. Ionic Liquid Crystal Elastomers for Actuators, Sensors, and Organic Transistors. Liq. Cryst. 2023, 50, 1151–1161. 10.1080/02678292.2023.2188615.

[ref33] Feng C.; Rajapaksha C. P. H.; Cedillo J. M.; Piedrahita C.; Cao J.; Kaphle V.; Lüssem B.; Kyu T.; Jákli A. Electroresponsive Ionic Liquid Crystal Elastomers. Macromol. Rapid Commun. 2019, 40 (19), 1900299 10.1002/marc.201900299. 31348584

[ref34] Khodagholy D.; Rivnay J.; Sessolo M.; Gurfinkel M.; Leleux P.; Jimison L. H.; Stavrinidou E.; Herve T.; Sanaur S.; Owens R. M.; Malliaras G. G. High Transconductance Organic Electrochemical Transistors. Nat. Commun. 2013, 4, 2133 10.1038/ncomms3133. 23851620 PMC3717497

[ref35] Alyami A.; Rajapaksha C. P. H.; Paudel P. R.; Kaphle V.; Kodikara S. G.; Lüssem B.; Jákli A. Bending Sensor Using Ionic Liquid Crystal Elastomers as Solid Electrolyte of Organic Electrochemical Transistors. Liq. Cryst. 2024, 51 (2), 297–304. 10.1080/02678292.2023.2297269.

[ref36] Rivnay J.; Leleux P.; Ferro M.; Sessolo M.; Williamson A.; Koutsouras D. A.; Khodagholy D.; Ramuz M.; Strakosas X.; Owens R. M.; Benar C.; Badier J. M.; Bernard C.; Malliaras G. G. High-Performance Transistors for Bioelectronics through Tuning of Channel Thickness. Sci. Adv. 2015, 1 (4), 1400251 10.1126/sciadv.1400251. PMC4640642 26601178

[ref37] Ortiz-Conde A.; García Sánchez F.; Liou J.; Cerdeira A.; Estrada M.; Yue Y.; Yue Y. A Review of Recent MOSFET Threshold Voltage Extraction Methods. Microelectron. Reliab. 2002, 42, 583–596. 10.1016/s0026-2714(02)00027-6.

[ref38] Inal S.; Malliaras G. G.; Rivnay J. Benchmarking Organic Mixed Conductors for Transistors. Nat. Commun. 2017, 8 (1), 1767 10.1038/s41467-017-01812-w. 29176599 PMC5701155

[ref39] Rivnay J.; Inal S.; Collins B. A.; Sessolo M.; Stavrinidou E.; Strakosas X.; Tassone C.; Delongchamp D. M.; Malliaras G. G. Structural Control of Mixed Ionic and Electronic Transport in Conducting Polymers. Nat. Commun. 2016, 7, 11287 10.1038/ncomms11287. 27090156 PMC4838877

[ref40] Friedlein J. T.; McLeod R. R.; Rivnay J. Device Physics of Organic Electrochemical Transistors. Org. Electron. 2018, 63, 398–414. 10.1016/j.orgel.2018.09.010.

[ref41] Doris S. E.; Pierre A.; Street R. A. Dynamic and Tunable Threshold Voltage in Organic Electrochemical Transistors. Adv. Mater. 2018, 30 (15), 1706757 10.1002/adma.201706757. 29498110

[ref42] Shahi M.; Le V. N.; Alarcon Espejo P.; Alsufyani M.; Kousseff C. J.; McCulloch I.; Paterson A. F. The organic electrochemical transistor conundrum when reporting a mixed ionic–electronic transport figure of merit. Nat. Mater. 2024, 23, 2–8. 10.1038/s41563-023-01672-4. 37880535

[ref43] Ohzono T.; Katoh K.; Fukuda J. I. Fluorescence Microscopy Reveals Molecular Localisation at Line Defects in Nematic Liquid Crystals. Sci. Rep. 2016, 6, 36477 10.1038/srep36477. 27812045 PMC5095605

[ref44] Jadzyn J.; Kêdziora P. Anisotropy of Static Electric Permittivity and Conductivity in Some Nematics and Smectics A. Mol. Cryst. Liq. Cryst. 1987, 145 (1), 17–23. 10.1080/00268948708080210.

[ref45] Harth K.; Stannarius R. Topological Point Defects of Liquid Crystals in Quasi-Two-Dimensional Geometries. Front Phys. 2020, 8, 112 10.3389/fphy.2020.00112.

