# Supplementary material for: Performance of Organic Electrochemical Transistors with Ionic Liquid Crystal Elastomers as Solid Electrolytes
Source: ACS Appl Mater Interfaces. 2024 Sep 26;16(40):54283–92. doi: 10.1021/acsami.4c06608 (PMC11472256; doi:10.1021/acsami.4c06608)
Supplement: Supplementary file 1 — am4c06608_si_001.pdf [file am4c06608_si_001.pdf]

Supporting Information

Performance of Organic Electrochemical Transistors with Ionic Liquid Crystal Elastomers as Solid Electrolytes

Arwa Alyami [1,2], Michael Skowrons[1], Kelum Perera [1,2] , Björn Lüssem[3,4,] and Antal Jákli [1,2]*

[1]Department of Physics, Kent State University, Kent OH, 44242, USA

[2]Advanced Materials and Liquid Crystal Institute, Kent State University, Kent OH, 44242, USA

[3]Institute for Microsensors, Microactuators, and Microsystems (IMSAS), University of Bremen, Bremen 28359, Germany

[4]MAPEX Center for Materials and Processes, University of Bremen, 28359 Bremen, Germany

*: Corresponding author: BLuessem@imsas.uni-bremen.de

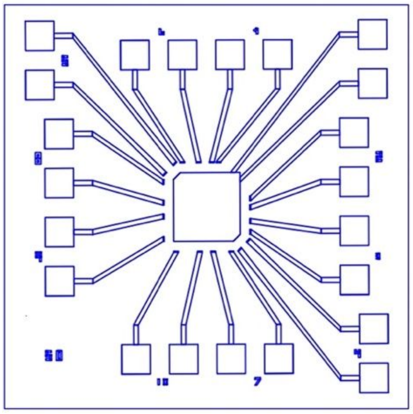

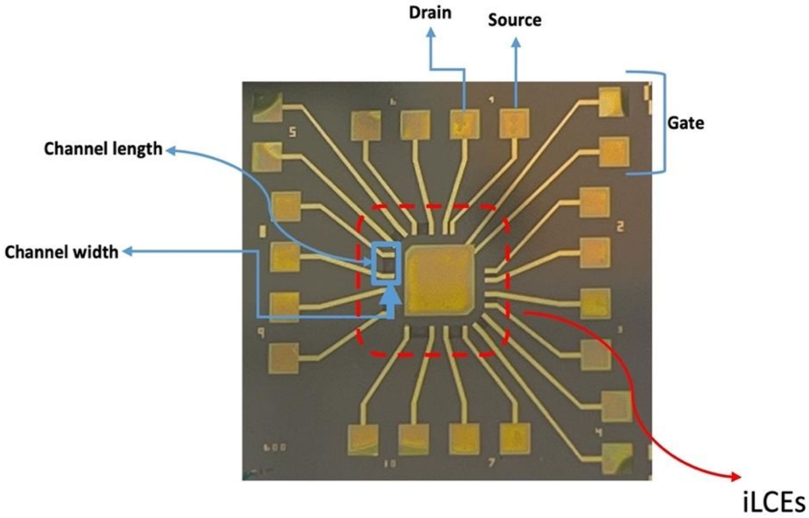

Figure S1: (left) Mask layout of the Si chip before deposition. The channel length of this device geometry is varied from 100 µm to 1000 µm and the width for each chip was varied from 50 µm to 600 µm, (right) The Si chip after deposition.

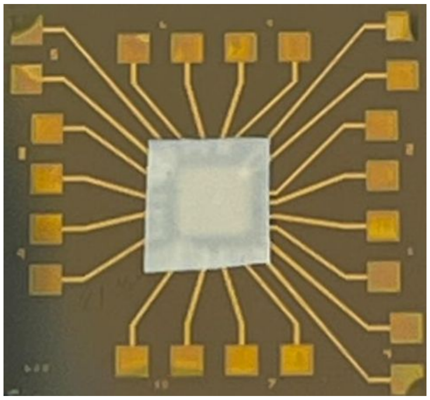

Figure S2: The ionic liquid crystal elastomer (iLCE) as solid electrolyte on the top of the OECT.

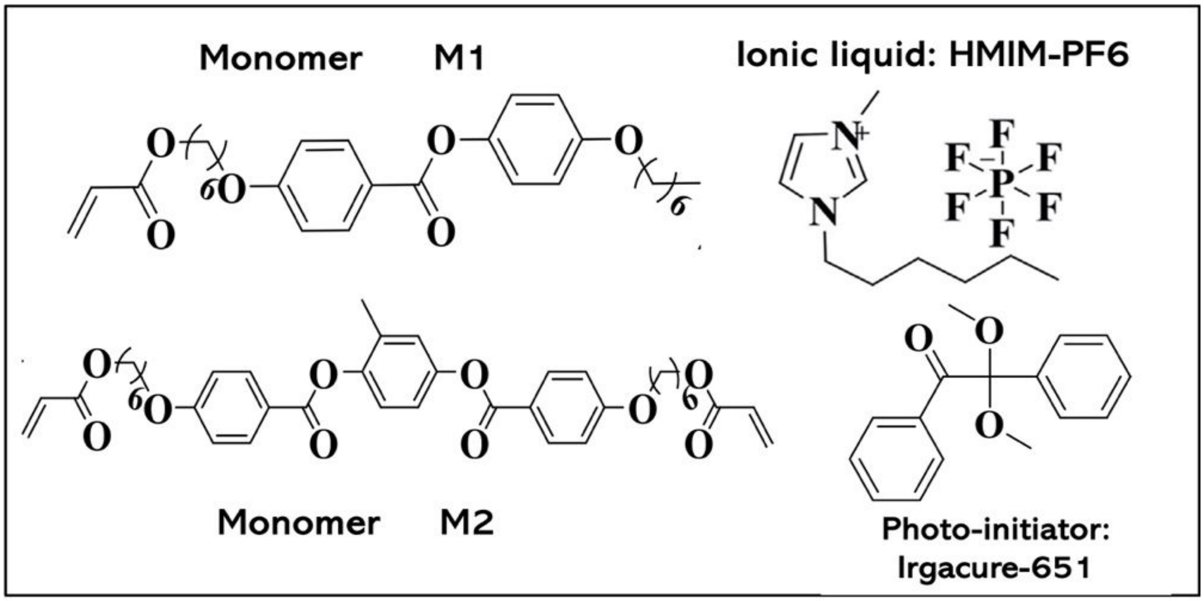

Figure S3: Molecular structures of substances used to make iLCE films. The ionic liquid HMIM-PF6 : (1-hexyl-3-methylimidazolium hexafluorophosphate), M1 (4-(6-Acryloxy-hex-1-yl-oxy) phenyl-4-(hexyloxy) benzoate) and M2 (1,4-Bis- [4-(6- acryloyloxyhexyloxy) benzoyloxy]-2-methylbenzene) are monofunctional and bifunctional mesogenic units respectively.
